# Supplementary material for: The Energetic Cost of Adrenergic Signaling in Primary Human Fibroblasts
Source: bioRxiv. 2026 Jul 10:2026.07.09.737569. Preprint. [Version 1] doi: 10.64898/2026.07.09.737569 (PMC13370414; doi:10.64898/2026.07.09.737569)
Supplement: Supplement 1 [file media-1.pdf]

Supplemental Figure 1

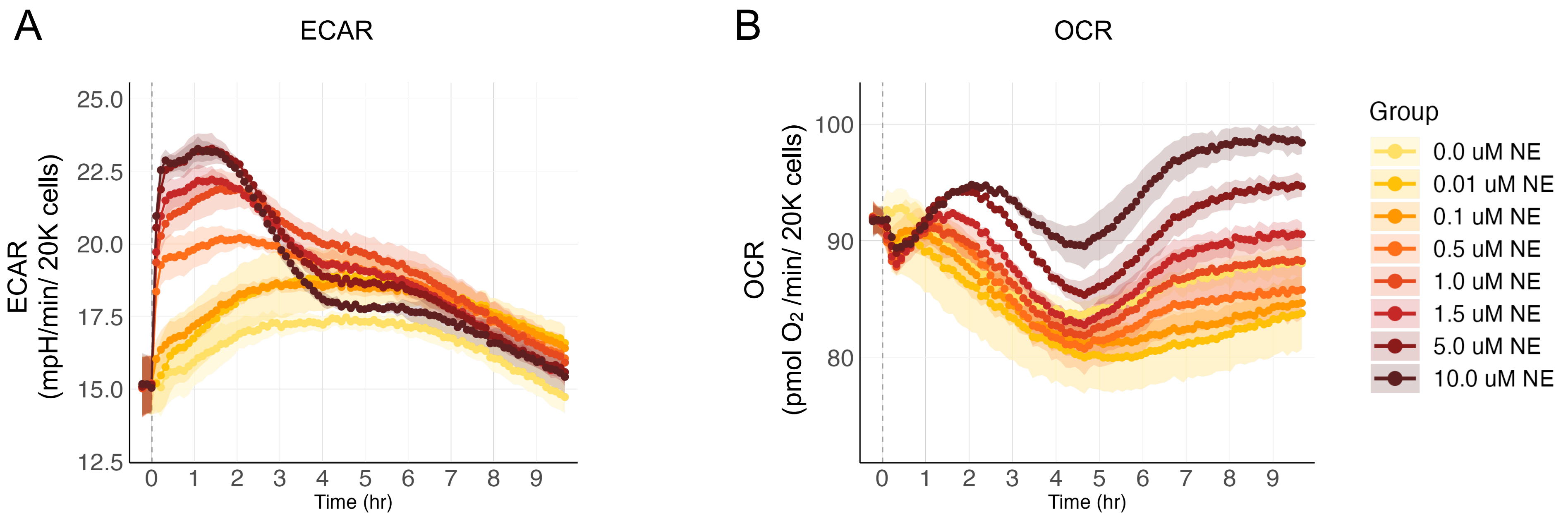

Supplemental Figure 1. Raw values of ECAR and OCR to NE doses over 10 hours (**A,B**) Raw average baseline-corrected values of ECAR and OCR of three separate 10 hour time-course experiments of fibroblasts in response to increasing doses of NE (0.0 to 10 μM NE). (**A**) extracellular acidification rate (ECAR) or (**B**) oxygen consumption rate (OCR). Vertical dashed line represents timing of NE or vehicle control injection. Wells per condition per seahorse experiment n = 8-12, 20K cells per well, individually run experiments n = 3.
