# Supplementary material for: The Energetic Cost of Adrenergic Signaling in Primary Human Fibroblasts
Source: bioRxiv. 2026 Jul 10:2026.07.09.737569. Preprint. [Version 1] doi: 10.64898/2026.07.09.737569 (PMC13370414; doi:10.64898/2026.07.09.737569)
Supplement: Supplement 3 [file media-3.pdf]

Supplemental Figure 3

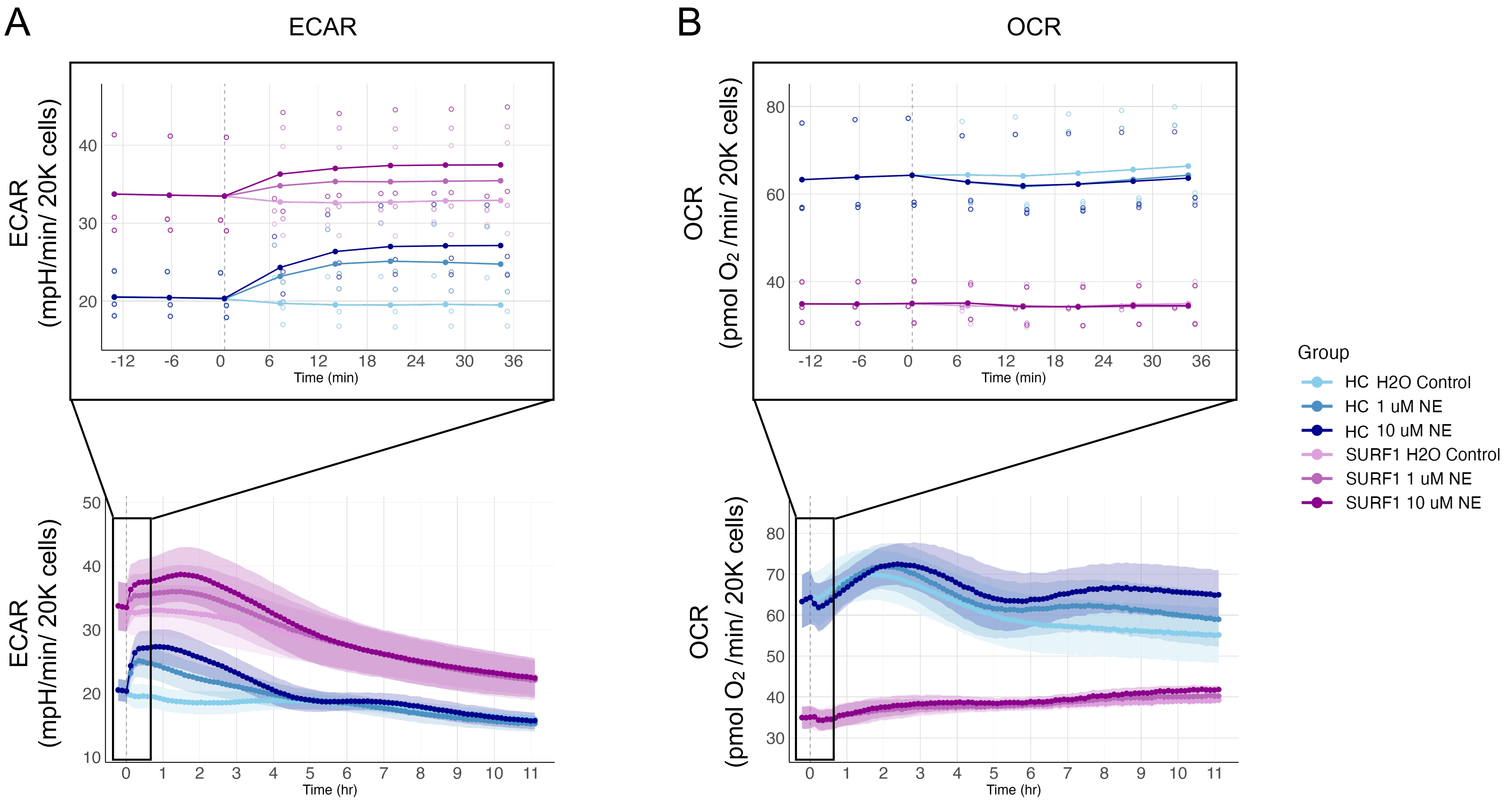

Supplemental Figure 3. Raw values for healthy control and SURF1 mutant time-courses (**A**) Time-course extending to 6 hours post NE injection of healthy control (HC) cells vs SURF1 mutant cells ± NE, as well as zoomed in on early timepoints (inset). Baseline-corrected to the three timepoints before the NE injection (**A**) Extracellular acidification rate (ECAR) (**B**) Oxygen consumption rate (OCR). Vertical dashed line represents timing of NE or vehicle control injection. Wells per condition per seahorse experiment n = 6-10, 20K cells per well, individually run experiments n = 3.
